# Obliterative Bronchiolitis in Workers in a Coffee-Processing Facility — Texas, 2008–2012

**Published:** 2013-04-26

**Authors:** Sharon Huff, James M. Stocks, Rena Saito, Patty Bilhartz, Jeffrey Levin, Craig Glazer, Rachel Bailey, Kristin Cummings, Kathleen Kreiss, Anna-Binney McCague

**Affiliations:** Depts of Occupational Health Sciences and Medicine, Univ of Texas Health Science Center at Tyler; Dept of Internal Medicine, Univ of Texas Southwestern Medical Center at Dallas; Div of Respiratory Disease Studies, National Institute for Occupational Safety and Health; EIS Officer, CDC

Obliterative bronchiolitis, a rare, irreversible form of fixed obstructive lung disease, has been identified in workers exposed to flavoring chemicals while working in the microwave-popcorn and flavoring-manufacturing industries (1); the occupational risk to workers outside these industries is largely unknown. This report describes two cases of obliterative bronchiolitis identified in workers employed in a small coffee-processing facility. Both patients’ illness was misdiagnosed before they received a diagnosis of work-related obliterative bronchiolitis, which had not been identified previously in the coffee-processing industry. These cases reinforce the need for exposure evaluation in all industries in which workers are exposed to flavoring chemicals. Additionally, a high index of suspicion is required when these potentially exposed workers have progressive shortness of breath. If obliterative bronchiolitis is suspected, immediate protection from further exposure is crucial to prevent further deterioration of lung function.

## Case Reports

### Patient 1

In October 2007, a nonsmoking, previously healthy Hispanic woman aged 34 years began work at the coffee-processing facility. Initially hired to work in the quality control laboratory, after 3 months she moved briefly to housekeeping, and then to the flavoring room. There, whole roasted coffee beans were mixed with liquid flavorings in an open process, ground, and packaged. Her primary tasks included operating the grinding and packing machines for these flavored coffee beans. After 1 year in this room, in January 2009, she transferred to a similar job in the unflavored coffee area, and in October 2011, she was dismissed.

The woman first sought care in November 2008, approximately 1 year after beginning work at the facility. She reported cough, shortness of breath on exertion, and occasional wheezing, which did not improve when away from work. Additional concerns included fatigue, throat dryness, constant thirst, and vertigo. Initial lung function testing showed severe obstruction responsive to bronchodilators (Table). She was hospitalized, and upon discharge was placed on antihistamines, inhaled steroids, and bronchodilators for possible asthma.

Despite initial improvement, 1 year later the woman visited a pulmonologist, describing worsening symptoms. Workup included repeat lung function testing, which demonstrated a worsening obstructive defect. Inspiratory and expiratory high-resolution computed tomography (HRCT) of the chest showed diffuse bronchial wall thickening, a prominent mosaic pattern, mild cylindrical bronchiectasis, and a small amount of fibrotic upper lobe scarring. Although inhaled steroids and mucus clearance therapy improved her cough, her dyspnea continued to worsen; an open lung biopsy was performed, which revealed constrictive bronchiolitis (the histopathologic correlate of obliterative bronchiolitis) with both narrowed and obliterated airways with surrounding fibrous tissue and a variable mixed chronic inflammatory cell infiltrate. Based on this result, she received a diagnosis of obliterative bronchiolitis.

At the patient’s most recent evaluation in April 2012, she continued to describe symptoms of severe shortness of breath with even light exertion, paroxysmal cough, and an inability to tolerate smells. Lung function testing at that time showed continued air trapping and severe obstruction marginally responsive to bronchodilators, and HRCT demonstrated disease progression. The patient currently is awaiting a lung transplant.

### Patient 2

In October 2009, a previously healthy, nonsmoking, Hispanic man aged 39 years went to work at the coffee-processing facility as an unloader, removing sacks of green coffee beans from trucks. Over the next 3 months he moved to maintenance, and then to the flavoring room, where he worked as a mixer. His job involved open bench-top weighing of liquid flavorings, which he poured into barrels of roasted coffee beans. A machine rotated these open barrels while he stood nearby to monitor the process. He worked there for about 19 months before moving to become a packer for unflavored coffee until placed on medical leave in 2012.

The man first noticed symptoms in April 2011, after working at the company for about 18 months. Although his initial concern was dyspnea with heavy exertion, he soon became short of breath with moderate activity. He received a diagnosis of bronchitis and was treated with steroids without significant improvement. He was subsequently placed on nasal and oral steroids, his workup failed to identify an allergic etiology, and he was referred to a pulmonologist in December 2011.

Although no longer working with flavorings, the man continued to describe cough, weight loss, and irritated eyes. Spirometry revealed severe obstruction. After 3 weeks of treatment with inhaled steroids, further testing confirmed this finding, and additionally demonstrated air trapping and a lack of bronchodilator response. HRCT of the chest showed a subtle mosaic abnormality with marked and diffuse air trapping, a few scattered centrilobular nodules, bronchial wall thickening, and mild bilateral cylindrical bronchiectasis. Consequent open lung biopsy revealed chronic and subacute small airways injury morphologically consistent with constrictive bronchiolitis. His doctors diagnosed his illness as obliterative bronchiolitis. At follow-up in May 2012, the patient said that although his cough had improved, his shortness of breath with exertion was worsening, and he was troubled by fatigue in the evenings.

In addition to their youth and shared work environment, these patients have much in common. Both initially had cough and dyspnea on exertion; their illness initially was misdiagnosed, and they were unsuccessfully treated with steroids and bronchodilators. In each case, a diagnosis of work-related obliterative bronchiolitis was made on the basis of lung function testing showing obstruction and hyperinflation, supportive HRCT and lung biopsy findings, and the temporal relationship between symptom onset and work exposure.

### Editorial Note

These two cases of obliterative bronchiolitis in a coffee-processing facility suggest expansion of the number of workers potentially at risk for flavoring-chemical related disease. They raise concerns about the current adequacy of identification of at-risk workers and workplace controls, and about possible underreporting of disease. Especially of note is the short employment tenure of affected workers and their apparent rapid decline in lung function. Although these patients were symptomatic within <18 months of work, their illness initially was unrecognized, leading to a diagnostic delay of 8–14 months. This is consistent with the natural history of obliterative bronchiolitis, which differs significantly from much chronic obstructive lung disease, where decline is slow and risk factors more apparent. Despite these differences, obliterative bronchiolitis often is misdiagnosed in such workers as asthma or chronic obstructive pulmonary disease, and therefore might be underreported.

CDC currently is evaluating health hazards at this facility to identify other potential cases, understand occupational exposures, and prevent new cases. Diacetyl, implicated as the cause of obliterative bronchiolitis in workers exposed to flavorings (2,3), was present in this workplace, according to some material safety data sheets accompanying flavoring materials to which affected workers were exposed. Yet other flavorings might have contained undeclared diacetyl because material safety data sheets only must mention recognized hazards of components comprising ≥1% of a product (4). Therefore, exposure assessment is necessary in this plant. Additionally, one substitute for diacetyl used in this facility has shown toxicity similar to diacetyl in laboratory animals (5).

Currently, no specific federal regulations govern workers exposed to diacetyl or its substitutes. CDC has drafted a recommended standard for occupational exposure that provides a quantitative risk assessment (6). One of the crucial recommendations, in addition to limitation of exposure, is regular hazard assessment in industries that use flavorings. Whereas most studies have focused on the microwave popcorn and flavoring industries, this report shows that other industries might benefit from the recommendations.

The findings in this report are subject to at least three limitations. First, the exposure of the two patients was not quantified; data on exposure of workers at the facility currently are being collected as part of the CDC health hazard evaluation. Second, diacetyl is produced by many foods, including coffee during the roasting process (7,8). Volatile organic compounds, including diacetyl, can be released during grinding (9). The relative contribution of diacetyl from flavorings and roasting or grinding to these two cases is unknown. Finally, production practices vary throughout the industry; therefore, it is possible this facility is not representative of other coffee-processing facilities.

Patients with a potential occupational exposure to flavoring chemicals should be considered at risk for obliterative bronchiolitis, and a high index of suspicion should be maintained. Because risk was not recognized previously in this industry, these two cases support the need for widespread hazard assessment in all industries using flavoring chemicals or generating diacetyl. For those patients suspected of having obliterative bronchiolitis, immediate intervention by removal from exposure is crucial to reducing respiratory morbidity and mortality.

What is already known on this topic?

Obliterative bronchiolitis is a severe, irreversible lung disease that can be caused by diacetyl in flavorings, as seen previously among workers in the microwave popcorn and flavoring production industries. The extent of obliterative bronchiolitis in other areas of the food industry is not known.

What is added by this report?

This report describes two cases of obliterative bronchiolitis in workers in a coffee-processing facility, an industry in which obliterative bronchiolitis had not been identified previously. Both patients experienced symptoms within <18 months exposure. Both have severe illness that was misdiagnosed for >8 months, and one is awaiting a lung transplant.

What are the implications for public health?

Obliterative bronchiolitis might be underdiagnosed in workers in the food and flavoring industries. The absence of recognized cases at facilities that use or produce flavoring chemicals does not mean absence of risk. Diagnosis requires a high index of suspicion, and early removal from the exposure is crucial to reducing respiratory morbidity and mortality from this irreversible disease.

## Figures and Tables

**TABLE:** Lung function test results for two coffee-processing workers with obliterative bronchiolitis, by month and year of test — Texas, 2008–2012

Test	Patient 1				Patient 2	
	
	Nov 2008	Dec 2009*	Feb 2010	Apr 2012	Dec 2011	Dec 2011
FVC % predicted	51	66	82	79	45	64
FEV1 % predicted	20	32	35	35	20	28
FEV1/FVC %	49	42	38	37	36	35
Bronchodilator response†	Yes	—§	No	Yes	—	No
TLC % predicted	117	134	—	—	—	111
RV % predicted	236	289	—	225	—	212
ERV % predicted	56	48	—	74	—	22
DLCO % predicted	Normal	Normal	—	Normal	—	Normal

**Abbreviations:** FVC = forced vital capacity; FEV1 = forced expiratory volume in 1 second; TLC = total lung capacity; RV = residual volume; ERV = expiratory reserve volume; DLCO = diffusing capacity of the lung for carbon monoxide.

* DLCO and lung volumes were performed 1 week after spirometry.

† Bronchodilator response was defined as a ≥12% change in FEV1 or FVC after bronchodilator administration.

§ Not reported.
